# Late-Onset Multiple Acyl-CoA Dehydrogenase Deficiency (MADD): Clinical Features, Diagnostic Challenges, and the Role of Oxidative Stress in Pathophysiology

**DOI:** 10.3390/antiox14121409

**Published:** 2025-11-26

**Authors:** Dario Zoppi, Anna Russo, Francesca Vallefuoco, Martina De Maria, Gabriella Esposito, Tiziana Fioretti, Valeria Maiolo, Filippo Maria Santorelli, Rosa Iodice, Stefano Tozza, Raffaele Dubbioso, Fiore Manganelli, Lucia Ruggiero

**Affiliations:** 1 Department of Neurosciences, Reproductive and Odontostomatological Sciences, University of Naples Federico II, 80131 Naples, Italy; anna.russo59@studenti.unina.it (A.R.); francesca.vallefuoco@unina.it (F.V.); martina.demaria@aslnapoli1centro.it (M.D.M.); rosa.iodice@unina.it (R.I.); fiore.manganelli@unina.it (F.M.); 2 CEINGE-Biotecnologie Avanzate Franco Salvatore, 80145 Naples, Italy; gabriella.esposito@unina.it (G.E.);; 3 Department of Molecular Medicine and Medical Biotechnologies, University of Naples Federico II, 80131 Naples, Italy; 4 Molecular Medicine, IRCCS Fondazione Stella Maris, 56128 Pisa, Italy; f.santorelli@fsm.unipi.it

**Keywords:** late-onset multiple acyl-CoA dehydrogenase deficiency, metabolic myopathy, glycogen storage disease, muscle biopsy, genetics, acylcarnitines, inflammatory myopathy, Guillain–Barré syndrome, misdiagnosis, oxidative stress

## Abstract

Introduction. Multiple Acyl-CoA Dehydrogenase Deficiency (MADD) is an autosomal recessive metabolic disorder resulting from mutations in the genes that encode the electron transfer flavoprotein (ETF) or its associated dehydrogenase (ETFDH), resulting in defects in mitochondrial fatty acid oxidation and a broad range of clinical presentations, most notably in the form of muscle weakness; exercise intolerance; and, in some cases, life-threatening metabolic crises. Late-onset MADD represents the most common form of lipid storage myopathy, but its diagnosis can be elusive due to its varied and often nonspecific clinical symptoms and may resemble other neuromuscular conditions, like inflammatory myopathies or other peripheral neuropathies, complicating the diagnostic process and delaying appropriate treatment. Aims. This case series aims to provide additional insights into the clinical presentation of MADD, highlighting diagnostic challenges in differentiating metabolic myopathies and emphasizing the role of muscle biopsy in diagnosing these conditions. Results. We describe five clinical cases of patients who were diagnosed with MADD, their clinical manifestations, and the diagnostic processes undertaken to arrive at this diagnosis. Notably, three patients were initially misdiagnosed with inflammatory myopathy, and one was misdiagnosed with Guillain–Barré syndrome. The correct diagnosis was established following a muscle biopsy, which revealed characteristic findings consistent with lipid storage myopathy and prompted subsequent biochemical analyses and genetic testing that confirmed the diagnosis of MADD. Conclusions. MADD is an underdiagnosed condition, and the description of new patients with various clinical presentations could support the development of clinical tools to promptly recognize this disease and allow physicians to deliver the most appropriate and effective therapy protocol, with Riboflavin and Carnitine supplementations, avoiding inappropriate treatments. The muscle biopsy was essential for a correct diagnostic assessment.

## 1. Introduction

Multiple Acyl-CoA Dehydrogenase Deficiency (MADD), also known as Glutaric Acidemia type II (GAII), is an autosomal recessive disorder caused by pathogenic variants in the genes encoding the electron transfer flavoprotein (ETF) or its dehydrogenase (ETFDH). These variants lead to a deficiency in the mitochondrial electron transport chain, impacting on the oxidation of fatty acids and some amino acids [1,2]. ETF, which is composed of the two subunits ETF-alpha and ETF-beta (encoded, respectively, by *ETFA* and *ETFB* genes), accepts electrons from several acyl-CoA dehydrogenases involved in fatty acid oxidation and from amino acid and choline metabolism pathways. Electrons are then transferred to ETFDH, located in the inner mitochondrial membrane, and finally to coenzyme Q (ubiquinone) in the electron transport chain [3]. Ubiquinone also functions as an intracellular antioxidant by removing oxygen free radicals, as well as transporting electrons and protons within the electron transport chain and between the processes of β-oxidation and oxidative phosphorylation through Complex III. Several studies have highlighted that MADD is characterized by a reduction in the intracellular levels of coenzyme Q10, which causes mitochondrial dysfunction and an increased production of reactive oxygen species (ROS) [4,5].

MADD is a rare disease, and its prevalence in the general population is estimated to be 1-9/1,000,000 [www.orpha.net, accessed on 10 September 2025]. However, the prevalence of the late-onset type is not precisely known [1]. Its clinical presentation can be divided into three categories according to the severity and age of onset. Type I is characterized by a neonatal onset with metabolic decompensation and congenital defects that may include facial dysmorphism, an enlarged liver, brain and/or kidney malformations, and unusual genitalia; type II is characterized by a neonatal onset and metabolic decompensation, without congenital defects; and type III is usually mild, with a late onset and progressive or fluctuating muscle weakness and episodes of rhabdomyolysis and metabolic crises [3,4,6]. Late-onset (type III) MADD is the most common lipid storage myopathy, mainly caused by a deficiency of ETFDH [2]. Since ETFDH activity is essential for the Complex III activity in skeletal muscle [7], its deficiency leads to the disruption of mitochondrial function [2,7,8,9]. Pathogenic variants in the *ETFDH* gene account for 93% of late-onset MADD [10]. In contrast, pathogenic variants in *ETFA* and *ETFB* genes are relatively more common in individuals with a neonatal presentation of MADD and account for 5% and 2% of type I and type II cases, respectively [3].

The late-onset form of MADD is commonly characterized by muscle weakness, exercise intolerance, and/or muscle pain, although metabolic decompensation with episodes of vomiting, lethargy, nonketonic hypoglycemia, hyperammonemia, metabolic acidosis, and even rhabdomyolysis has also been reported. These symptoms are often triggered or exacerbated by stress conditions, like illnesses, exercise, or fasting. Patients may rarely develop sensory neuropathy symptoms, presenting with numbness of the extremities and sensory ataxia [1,3]. Most affected individuals develop chronic symptoms such as muscle weakness, fatigue, myalgia, and exercise intolerance, even though the most common myopathic presentation is progressive or fluctuating proximal myopathy. Progressive weakness may also involve respiratory muscles, leading to acute or subacute respiratory failure [3]. The diagnosis of late-onset MADD, which mainly involves adult subjects, can be particularly challenging due to its subtle and inconsistent presentation. It can occur in an acute or subacute fashion, mimicking other neuromuscular diseases such as inflammatory myopathies. Typically, laboratory tests detect elevations of several acylcarnitines in serum and an increased excretion of multiple organic acids in urine. A diagnosis is finally established by genetic testing, with the identification of biallelic pathogenic variants in *ETFA*, *ETFB*, or *ETFDH* genes [1,3]. The clinical management of late-onset MADD is primarily focused on symptom control and the prevention of metabolic crises. Dietary modifications, such as low-fat, high-carbohydrate diets supplemented with medium-chain triglycerides, are central to treatment. Riboflavin supplementation is particularly effective in patients with *ETFDH* mutations, as it can enhance residual enzyme activity. Carnitine supplementation is also commonly used to facilitate the removal of toxic acylcarnitines produced as a consequence of enzymatic deficiency [1].

Herein, we present five patients with late-onset MADD associated with pathogenic variants in the *ETFDH* gene, all characterized by an acute (peak of symptoms in less than 4 weeks) or subacute (peak of symptoms in 4 to 8 weeks) clinical onset. We describe their clinical features and the diagnostic pathway, focusing on the challenges of reaching a differential diagnosis that rules out other neuromuscular disorders with overlapping symptoms. We emphasize the pivotal role of muscle biopsies in guiding the diagnosis towards lipid storage myopathy and in confirming MADD through biochemical and/or genetic analyses.

## 2. Materials and Methods

Patients underwent complete clinical evaluation and neurological examination; blood tests, including the evaluation of serum amino acids; acylcarnitines; and a wide panel of organic acids involved in energy production, amino acid catabolism, and fatty acid oxidation—these included short-chain hydroxy and keto acids (lactic, 3-hydroxybutyric, 2-methyl-3-hydroxybutyric, and 2-keto-3-methylvaleric), reflecting glycolytic and branched-chain amino acid metabolism; dicarboxylic acids, such as succinic, fumaric, glutaric, adipic, pimelic, and azelaic acids, representative of the Krebs cycle and fatty acid ω-oxidation intermediates; methylmalonic/ethylmalonic-related compounds, including 2-methylsuccinic and 2-methyl-3-hydroxybutyric acids, indicative of alterations in short-chain fatty acid oxidation; and aromatic derivatives, such as 2-hydroxyphenylacetic, β-hydroxyphenylacetic, and benzoic acids, markers of aromatic amino acid metabolism. Targeted metabolomic analysis of acylcarnitines was performed on serum using tandem mass spectrometry (MS/MS), while urinary organic acids were extracted, derivatized, and analyzed via gas chromatography–mass spectrometry (GC–MS). Quantification relied on stable-isotope internal standards, allowing accurate detection of metabolites related to mitochondrial and fatty acid oxidation pathways [11,12]. Electrophysiological factors were assessed through nerve conduction and needle electromyography. Muscle magnetic resonance imaging (MRI) was also acquired. Muscle biopsy was performed, and cryostat sections with a thickness of 10 microns were processed with the following histological and histochemical methods: hematoxylin–eosin (HE), Gomori trichrome (TG), cytochrome c oxidase (COX), succinate dehydrogenase (SDH), combined COX-SDH (COMBI), nicotinamide adenine dinucleotide (NADH), ATP-ase at pH 4.3, 4.6, and 9.4, periodic acid–Schiff (PAS), Phosphorylase (FO), and Sudan Black. Genomic DNA (gDNA) was extracted from peripheral blood leukocytes according to standard procedures. Mutation analysis was performed by Next-Generation Sequencing (NGS), as previously described [13]. Library preparation was carried out using either the Twist Human Core Exome (Twist Bioscience, San Francisco, CA, USA) or the SureSelect Clinical Research Exome/SureSelect FocusedXT Exome CCP17 kits (Agilent Technologies, Inc; San Diego, CA, USA), enabling the analysis of ~4800–5000 disease-associated genes, including those related to neuromuscular disorders. Sequencing was performed on NextSeq500 or NovaSeq platforms (Illumina Inc., San Diego, CA, USA). Read alignment, variant calling, and annotation were performed using a validated in-house bioinformatics pipeline with high sensitivity for single-nucleotide variants (SNVs) and lower sensitivity for copy number variations (CNVs). Variant prioritization was guided by the patient’s phenotype, and pathogenicity was assessed according to the American College of Medical Genetics and Genomics (ACMG) criteria, starting from those indicated by Franklin (https://franklin.genoox.com, accessed on 11 September 2025). Human genome hg19 coordinates were used as reference. Identified variants were validated, and segregation analysis was performed using PCR and Sanger sequencing on family members’ DNA. Total RNA was extracted from peripheral blood leukocytes, and reverse transcriptase PCR (RT-PCR) was performed using 1 µg of RNA and primers spanning exons 10–12 of the *ETFDH* transcript; cDNA amplicons were subsequently analyzed via Sanger sequencing.

## 3. Results

**Figure 1:**
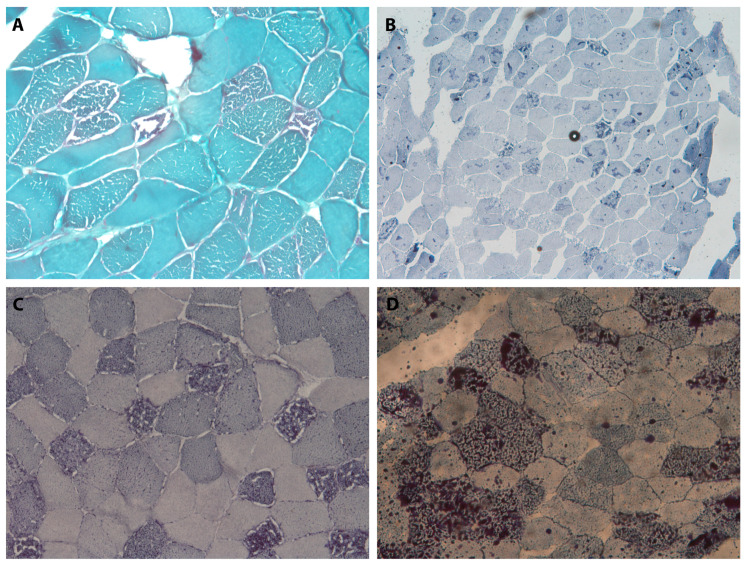
Muscle biopsy. (**A**) (patient 1): Gomori’s Trichrome staining (TG) showing fiber size variability and vacuolated fibers without inflammatory infiltrates. (**B**) (patient 2), (**C**) (patient 1) and (**D**) (patient 5): Sudan Black staining highlighting intracytoplasmic and subsarcolemmal vacuoles with marked lipid accumulation, consistent with lipid storage myopathy.

Table 1 summarizes the clinical, laboratory, instrumental, and genetic data of the reported patients; Figure 1 presents the images and data of the histopathological investigations.

### 3.1. Case 1

A 44-year-old male patient with a history of hypokinetic dilated cardiomyopathy with a moderate reduction in EF (45%) and normal coronary angiography came to our attention due to a subacute onset of lower limb weakness and diffuse myalgia. His family history was negative for neuromuscular or cardiac pathologies. The blood biochemical analysis demonstrated an increase in CPK (1800 U/L, n.v. 20–180 U/L), LDH (1516 U/L, n.v. 135–214 U/L), AST (109 U/L, n.v. 10–50 U/L), and ALT (83 U/L, n.v. 10–35 U/L), which was confirmed in further dosages. About eight months later, he developed a dropped head, fatigue, shortness of breath when walking for long distances, and poor exercise tolerance. He also reported involuntary weight loss (8 kg in five months). During his hospitalization he underwent autoimmune and viral infection screening, spirometry, dry blood spot (DBS) for alpha-glucosidase and alpha-galactosidase analyses, and an electrophysiological study, which all revealed normal results. Muscle MRI of the lower limbs showed bilateral edematous imbibition of the semimembranosus and biceps femoris muscles, a more marked and widespread Short Time Inversion Recovery (STIR) hyperintensity of the soleus, and a milder STIR of the posterior tibialis muscles, bilaterally. STIR hyperintensity is typically indicative of edema (increased water content) or myxoid changes within the muscle tissue, reflecting inflammation but also other conditions such as acute injury or denervation.

Based on these findings, he was diagnosed with polymyositis and treated with Prednisone at 62.5 mg per day. Due to a lack of response to the steroid therapy and the persistence of symptoms, a muscle biopsy was performed that revealed that more than 50% of the fibers, predominantly histotype 1, were affected by degenerative processes with diffuse vacuolization at both cytoplasmic and subsarcolemmal locations, which was apparent with the use of Sudan Black staining. A marked variability of fiber calibers with hypotrophic fibers was also noted. Due to these findings, a genetic investigation for metabolic myopathies was performed, which highlighted the presence of a homozygous variant, c.1204A > G (p.Thr402Ala), in the *ETFDH* gene. This variant met four ACMG pathogenicity criteria (PM2, PM1, PP2, and PM) and was classified as likely pathogenic (class 4). Accordingly, a diagnosis of MADD was established, and treatment with 100 mg of Riboflavin per day, 200 mg of Ubidecarenone (Q10 coenzyme) per day, and 1 g Carnitine supplementation per day was initiated. In the subsequent clinical evaluations, the clinical picture improved significantly. Furthermore, CPK and LDH levels returned to normal values. Then, Prednisone therapy was slowly tapered with sustained clinical benefits.

### 3.2. Case 2

Case 2 was a 37-year-old male patient with an insidious onset of slowly progressive easy tiredness and diffused myalgia with associated slight hyperCKemia (values between 400 and 500 U/L in different tests). A progressive increase in CPK values higher than 2000 U/L, associated with a slight increase in transaminases (AST and ALT), was detected. For these reasons, an electrophysiological study was carried out that showed a myopathic pattern with diffuse slight fibrillation activity (neurography was normal), and muscle MRI highlighted edematous alterations mainly at the level of the posterior compartment of the thigh and leg muscles. Spirometry was performed and yielded normal FVC/FEV1 ratios. Also, myositis-specific antibody screening was performed, which produced negative results. The patient was diagnosed with inflammatory myopathy and began therapy with 75 mg of Prednisone per day with a partial clinical improvement, moving from using a wheelchair in extra-domestic settings to walking with a cane. However, the symptoms did not completely resolve, and the CPK levels did not return to normal values. So, we decided to perform a muscle biopsy, which showed that approximately 30% of the fibers, predominantly histotype 1, were affected by degenerative processes, presenting significant vacuolization in multiple cytoplasmic and subsarcolemmal locations, in most cases, which were optically empty in Hematoxylin and Eosin (H&E) and Gomori’s Trichrome stains but strongly positive in Sudan Black stains, which is compatible with myopathy due to a lipid metabolism disorder without apparent inflammatory elements. Genetic investigations identified a heterozygous likely pathogenic variant in the *ETFDH* gene, c.1852T > C (p.Ter618Gln; PM3, PP3 PP5, class 4); no other variants were identified in the whole gene coding region. The c.1852T > C sequence change disrupted the translational stop signal of the *ETFDH* mRNA. It was expected to extend the length of the ETFDH protein by 13 additional amino acid residues. This protein extension has been observed in individuals with severe or lethal forms of Multiple acyl-CoA Dehydrogenase Deficiency [14,15]. The serum acylcarnitine profile was determined to support the diagnosis, which revealed a consistent increase in medium- and long-chained fatty acids from C5 to C18. The increased excretion of multiple organic acids in urine was also detected. Furthermore, cases of late-onset MADD associated with a single mutation had already been described in the literature [16]. Thus, we prescribed therapy with Riboflavin, 100 mg per day; Ubidecarenone, 200 mg per day; and Carnitine supplementation, 1 g per day, in addition to Prednisone. For this patient, we decided to continue the Prednisone therapy due to muscle inflammation demonstrated by electrophysiological tests and muscle MRI, which was further supported by the initial clinical response. In approximately three months, he reported a marked improvement in symptoms, and CPK levels returned to the normal range. After six months, the Prednisone therapy was tapered and discontinued.

### 3.3. Case 3

A 26-year-old female patient, in conjunction with an episode of acute pharyngitis, started to complain of diffuse asthenia. Symptoms gradually worsened, leading to the involvement of lower limb strength. An increase in CPK was identified, with values of 1100 U/L, with an associated increase in LDH and a slight increase in transaminases. She underwent an electrophysiological study, which highlighted a myopathic pattern with fibrillation activity, and a normal nerve conduction study. According to these findings, she was diagnosed with myositis and treated with 1000 mg Methylprednisolone boluses followed by Prednisone at 50 mg per day, with only a slight clinical improvement. During hospitalization, the patient underwent a spirometry analysis that showed a mild restrictive ventilatory deficit; a muscle MRI of the pelvis and lower limbs that displayed mild adipose infiltration of the gluteal muscles and posterior thighs bilaterally; negative myositis-specific antibody screening; and negative autoimmune screening. After approximately a year, a worsening of her symptomatology with an increase in weakness occurred. Also, an increase in CPK values over 2100 U/L and an increase in LDH to 1100 U/L were confirmed. Finally, she underwent a muscle biopsy, which revealed that approximately 40% of the fibers, predominantly histotype 1, were affected by degenerative processes and presented diffuse vacuolization, in most cases with multiple vacuoles with both cytoplasmic and subsarcolemmal locations, which was strongly demonstrated by the Sudan Black staining. A moderate variability of fiber calibers with hypotrophic fibers was also noted. Genetic analyses detected a compound heterozygous genotype in the *ETFDH* gene due to the variants c.358G > C (p.Asp120His) (PM2, PP2, PM2, and PM3, likely pathogenic, class 4), inherited from the mother, and c.814G > A (p.Gly272Arg) (pathogenic, class 5), inherited from the father. She began therapy with Riboflavin, at 100 mg per day; Ubidecarenone, at 200 mg per day; and a Carnitine supplementation, at 1 g per day, with a significant clinical improvement. CPK and LDH levels returned to the normal range.

### 3.4. Case 4

Case 4 involved a subacute onset of easy fatigability and the proximal weakness of lower limbs in a 27-year-old male patient; symptoms began following an episode of acute gastroenteritis with fever and diarrhea. Blood tests revealed hyperCKemia, with values greater than 1200 U/L. The patient also reported an intolerance to fasting with a feeling of malaise and tiredness. His parents were first cousins. He underwent an electrophysiological study that highlighted a diffuse myopathic pattern. Subsequently, we performed a muscle biopsy that pinpointed that more than 40% of the fibers, predominantly histotype 1, were affected by degenerative processes presenting numerous vacuolizations, in most cases multiple and with both cytoplasmic and subsarcolemmal locations, that were strongly positive in the Sudan Black staining. A genetic analysis revealed a homozygous intronic variant (c.1286-15T > A) in intron 10 of the *ETFDH* gene, which was classified as a variant of uncertain significance (VUS) (PM2, class 3) in the bioinformatic analysis. A segregation study identified the variant in a heterozygous state in the consanguineous parents. This variant was further investigated through a functional study of the mRNA, which confirmed its pathogenicity. Indeed, the RT-PCR performed on the mRNA region spanning exon 10–12 produced two cDNA fragments of different lengths. Sanger sequencing revealed the skipping of exon 11 in the shorter mRNA isoform, which led to a loss of function, whereas the longer fragment had a normal sequence. The patient promptly began therapy with Riboflavin at 100 mg per day, Ubidecarenone at 200 mg per day, and Carnitine supplementation at 1 g per day, with a complete resolution of symptoms and a normalization of CPK values.

### 3.5. Case 5

A 34-year-old female patient complained of a subacute onset of pain localized to lower limbs in an ascending fashion, which was quickly accompanied by weakness involving lower limbs and then the shoulder girdle as well. Approximately two weeks earlier, the patient had gastroenteritis with fever and diarrhea. She also complained of hypoesthesia and paresthesia in distal extremities of her upper and lower limbs. For these reasons, before beginning our observations, she began therapy with Gabapentin. In the same period, the patient reported marked weight loss (about 20 kg), apparently not related to changes in her lifestyle or diet, and episodic diarrhea. Due to the persistence of neurologic symptomatology, she was admitted to our department. During hospitalization, she underwent the following tests: a normal blood chemistry screening; an electrophysiological study that showed essentially a sensory neuropathy mainly affecting the lower limbs; spinal MRI, which showed no significant alterations; a lumbar puncture that showed only a mild increase in the CSF protein level of 63 mg/dL (n.v. 15–45 mg/dL); negative screening for celiac disease; negative viral infection screening; and autoimmune screening that showed a weak positivity of the anti-GM2 antibody. As such, in consideration of the clinical picture, the positive history of gastroenteritis prior to the symptom onset, and the identification of the anti-GM2 antibody positivity, she was treated with IVIG with an uncertain benefit and discharged with a diagnosis of Guillain–Barré Syndrome (GBS). After approximately one year, she experienced a worsening of symptoms, which forced her to use a wheelchair in extra-domestic settings. Blood tests revealed an increase in serum CPK (700 U/L). As such, we performed a muscle MRI, which highlighted a partial fibroadipose replacement of the thigh muscles, and a new electrophysiological study, which showed a myopathic pattern with a reduction in the duration of motor unit potentials (MUPs) without spontaneous muscular electrical activity, associated with axonal sensory neuropathy and with a reduction in the SNAP (Sensory Action Potential) amplitude in the lower limbs. For these reasons, a muscle biopsy of the biceps brachii was performed. The histological investigation revealed that approximately 40% of the fibers, predominantly histotype 1, were affected by degenerative processes presenting diffuse vacuolization, in most cases with multiple vacuoles with both cytoplasmic and subsarcolemmal locations, optically empty in the H&E and Gomori’s Trichrome stains but strongly apparent in the Sudan Black stains, indicating intrafiber lipid accumulation and, therefore, muscle damage due to the alteration of lipid metabolism. In addition, we performed an NGS genetic analysis, which identified a heterozygous pathogenetic splicing variant in *ETFDH*, namely c.406-2A > G [17]; no other nucleotide changes were detected in the whole gene coding region. As MADD was suspected, we assessed the serum acylcarnitine profile, which revealed a consistent increase in medium- and long-chained fatty acids from C5 to C18; an increased urinary excretion of multiple organic acids was also detected. We began treatment with Riboflavin, 100 mg per day; Ubidecarenone, 200 mg per day; Carnitine supplementation, 1 g per day; and Prednisone, 25 mg per day. Contextually, the patient began physiotherapy and experienced a significant improvement in symptoms within a few months. She went from using a wheelchair in extra-domestic environments to walking independently and recovered almost all autonomy in her daily activities. We also monitored serum CPK, and when it returned to normal values, the Prednisone therapy was tapered and discontinued. In subsequent clinical evaluations, the patient’s neuromuscular symptoms continued to be stable.

## 4. Discussion

In this study, we described five patients who had a clinical diagnosis of MADD. Every patient had an acute/subacute onset in their third or fourth decade, reaching their symptom peak within a few weeks. In four out of five patients, it was possible to identify a trigger event, such as gastrointestinal, respiratory infections, or even acute coronary syndrome. Triggering events usually occurred within two weeks prior to the symptom onset. Two patients (Cases 1 and 5) also reported significant weight loss preceding the onset of their symptoms, apparently unrelated to the disease, which may have been a contributing factor, together with the triggers, of the acute exacerbation of symptoms. No clear regain of body weight was observed following the replacement therapy. Notably, in the patient without any recognizable trigger event (Case 2), symptoms developed more slowly, over weeks or months. The clinical presentation was predominantly muscular in all patients, with exercise intolerance and proximal weakness especially involving lower limbs. Diffuse myalgia was also a very common symptom. One of the patients developed a dropped head, and another one reported a history of intolerance to fasting with a subsequent feeling of general malaise and nausea. Only one out of our patients showed a peculiar clinical picture, as she developed a proximal weakness of her lower limbs preceded by pain and paresthesia of distal extremities, which seemed to be the dominant symptom in the initial days. Regarding blood tests, all patients displayed hyperCKemia, with values up to 1800 U/L, together with mild hypertransaminasemia and a consistent increase in serum LDH levels (more than we are used to seeing in other myopathies or myositis). Electrophysiological tests demonstrated, in most cases, myopathic alterations in association with fibrillation activity; the latter was more evident in some patients and was less evident in others. Nerve conduction studies were normal, except for Case 5, in which we detected a predominantly sensory axonal neuropathy. Two patients underwent lower limb muscle MRI, which, in both cases, demonstrated muscular edema associated with STIR hyperintensities. From a histopathological point of view, all the muscle biopsies identified fiber degeneration, especially type I, with the presence of multiple vacuoles located both in cytoplasmic and subsarcolemmal regions. These same fibers demonstrated lipid accumulation, evidenced by their positivity to Sudan Black staining, with a marked hyperactivity of vacuoles. Other stains revealed no peculiar or specific histopathological changes. While mitochondrial dysfunction is present in these conditions, no clear abnormalities of oxidative enzyme activities were demonstrated in the NADH, COX, SDH, or COMBI histochemistry. In our patients, a muscle biopsy was essential to distinguish metabolic from inflammatory myopathies. Nevertheless, in lipid storage myopathies, biopsy findings may range from evident lipid accumulation within vacuolated fibers to completely normal morphologies, especially in mild or intermittent cases; therefore, a normal biopsy cannot confirm diagnoses.

Genetic investigations, performed via NGS targeting a large panel of genes associated with metabolic myopathies, detected *ETFDH* gene mutations in all patients. Two patients possessed homozygous pathogenic variants: one was a compound heterozygote, and in two patients only monoallelic pathogenetic variants were identified. Notably, several cases of late-onset MADD with only one mutated allele have been reported [16], likely due to missing heritability problems rather than a semi-dominant effect of the variant. Indeed, late-onset MADD represents the milder form of a severe genetic metabolic disorder, and, in agreement, it is associated with a partial deficiency of the ETFDH enzyme activity, as in Case 4, who was homozygous for the c.1286-15T > A intronic variant that strongly reduced, but did not abolish, the normal RNA splicing. In contrast, the monoallelic variant c.1852T > C (p.Ter618Gln) we found in Case 2 was previously identified in patients affected by very severe or lethal forms of the disease [13,14], similarly to the c.406-2A > G splicing variant (Case 5), which, by affecting the acceptor splice site in intron 3 of the *ETFDH* gene, is expected to disrupt RNA splicing. Indeed, variants that disrupt the donor or acceptor splice site typically lead to a loss of protein function [18], and loss-of-function variants in *ETFDH* are known to be pathogenic and associated with severe neonatal phenotypes [19]. Therefore, we hypothesize that these patients could possess a hypomorphic variant on the other allele that partially reduces the activity or amount of the functional enzyme. Unfortunately, an RNA analysis, which could have identified the possible other variants, was not performed for these two patients.

All patients’ diagnosis was confirmed via the clinical presentation and histopathological findings as well as serum acylcarnitine testing, which showed an increase in medium- and long-chained fatty acids from C5 to C18 [20]. In addition, urinary organic acid analyses revealed increased concentrations of lactic, glycolic, glyoxylic, pyruvic, succinic, methylsuccinic, and glutaric acids, while citric and hippuric acids were decreased compared to controls. Although this metabolic profile is not highly specific, it is consistent with that typically observed in late-onset ETFDH deficiency (Multiple acyl-CoA Dehydrogenase Deficiency), characterized by the accumulation of dicarboxylic acids and secondary mitochondrial dysfunction. These assessments were carried out both before and after the specific therapy. It is important to note that normal serum acylcarnitine and urinary organic acid profiles can be observed in MADD, especially in milder or episodic cases, and that biochemical normalization does not always reflect clinical recovery. For further diagnostic confirmation, in addition to the biochemical tests, enzyme assays testing fatty acid oxidation (FAO) defects in patients’ skin fibroblasts should also be taken into account; however, none of our patients provided consent for skin fibroblast sampling. In addition, a diagnostic confirmation was obtained from the quite fast clinical response to therapy with Riboflavin plus Ubidecarenone (Q10 coenzyme) and Carnitine integration, which induced a complete resolution of muscular symptoms and the normalization of CPK and LDH levels, in all patients [21]. We must underline that at the follow-up carried out approximately 3 years later, the patient (Case 5) who presented with an overlapping sensory neuropathy continued to exhibit symptoms consistent with paresthesia and numbness of the lower extremities and electrophysiological findings compatible with an axonal sensory neuropathy, demonstrated by the reduction in the SNAP amplitude in lower limbs [22,23].

It should be noted that three out of five patients were initially misdiagnosed with inflammatory myopathy, presumably because both the clinical (subacute onset, worsening proximal weakness, and muscle pain) and laboratory instrumental findings (blood tests, electrophysiology, and muscle MRI) mimicked polymyositis. For this reason, it should be considered in the differential diagnostic of these forms [24,25]. Other cases of MADD misdiagnosed as inflammatory myopathy have been previously described [10,16,23,26,27,28,29,30,31]. Moreover, patients who initially received a diagnosis of inflammatory myopathy were treated with Prednisone-based steroid therapy and showed partial, although not lasting, responses to anti-inflammatory therapy.

Considering all these factors, we considered that MADD, in its acute presentation, can resemble and mimic an inflammatory muscle pathology that, moreover, seems to respond to steroid therapy, although the reported benefit from such treatments is limited or absent. Conversely, it has been observed that symptoms effectively resolve with a supplementation of Riboflavin, Ubidecarenone, and Carnitine; the latter must be administered with caution and under strict cardiologic surveillance due to the potential risk of cardiotoxicity. It is widely recognized that fibroblasts of MADD patients demonstrate an increased production of reactive oxygen species (ROS) and oxidative stress, as a consequence of two main mechanisms, namely a reduced synthesis of coenzyme Q10, a known ROS scavenger and powerful antioxidant, and a direct leakage of electrons by the misfolded and deficient ETFDH protein, which is therefore unable to effectively transfer electrons to Complex III of the mitochondrial respiratory chain [4]. This suggests the existence of a link between ETFDH, Complex III, and QH2 (ubiquinol), which is essential for oxidative phosphorylation efficiency and CoQ homeostasis. The resulting chronic oxidative stress causes mitochondrial dysfunction, which is associated with increased mitophagy [32,33,34]. Mitophagy is a quality control mechanism that allows for the selective degradation of damaged mitochondria, thereby preventing further cellular dysfunction. Consequently, mitophagy dysfunction may culminate in myocyte apoptosis [4,8,35]. Furthermore, in the fatty acid oxidation disorder closely related to MADD, the very-long-chain acyl-CoA dehydrogenase deficiency (VLCADD)—the accumulation of upstream products from dysfunctional beta-oxidation, such as free fatty acids (FFAs) and acylcarnitines—is responsible for altered cellular signaling that results in an overproduction of inflammatory cytokines [4,36].

Based on these findings, we can infer that the interaction between chronic oxidative stress, which is widely recognized in MADD patients due to reduced CoQ10 synthesis and direct electron leakage from the deficient ETF-QO protein, and the possibility of an altered cellular signaling, resulting in an overproduction of pro-inflammatory cytokines (as observed in VLCADD due to the accumulation of free fatty acids and acylcarnitines) [36], could induce an alteration in inflammatory homeostasis, leading to a pro-inflammatory fibrocellular state. Under conditions of increased metabolic demands, triggered by factors such as fever, infections, fasting, intense physical exercise, or intercurrent illnesses, this pre-existing state could be responsible for clinically significant muscle inflammation, which overlays the already known energy deficit characteristic of these pathologies. Further studies are needed to confirm these inferences, especially because, based on these considerations, we believe that a therapeutic cycle of corticosteroid therapy could be beneficial for this specific category of patients with acute/subacute muscular symptoms.

## 5. Limitations

This study has several limitations that should be taken into account when interpreting the results. The small number of included cases (five patients) limits the generalizability of the findings to a broader population, especially considering the marked clinical and genetic heterogeneity of Multiple Acyl-CoA Dehydrogenase Deficiency (MADD). Moreover, the retrospective nature of this case series may introduce a selection bias and is dependent on the completeness of the available clinical and laboratory data.

Another limitation concerns the fact that, particularly for patients carrying a single heterozygous mutation, no functional studies (such as enzymatic assays or protein expression analyses) were performed. These could have confirmed the pathogenic impact of the variant and helped to better define the genotype–phenotype correlation.

## 6. Conclusions

The acute clinical presentations described in this work could represent a typical manifestation of MADD and should be considered in the diagnostic workup of this condition. None of our patients experienced acute metabolic crises, acidosis, or myoglobinuria. All showed elevated serum CPK levels, sometimes exceeding 2000 U/L, along with disproportionately elevated LDH, a pattern suggestive of metabolic myopathy. Electro-physiological studies and muscle MRI, when performed, revealed findings that can mimic acquired inflammatory myopathies. Combined with the initial response to steroid therapy, this overlap can lead to diagnostic and therapeutic errors.

These observations suggest that some cases diagnosed as inflammatory myopathy without histological confirmation and with incomplete responses to immunosuppressive therapy may be undiagnosed MADD. This supports the idea that MADD remains underrecognized, and reports of new cases with variable phenotypes may contribute to its earlier diagnosis.

Ultimately, an accurate diagnosis is essential not only to begin targeted treatments, such as Riboflavin and Carnitine, but also to prevent the risks of prolonged, inappropriate therapies. This case series underscores the key role of muscle biopsies in distinguishing metabolic, inflammatory, and genetic myopathies with overlapping clinical features.

## Figures and Tables

**Table 1:** Summary table of demographics; clinical, instrumental, and laboratory diagnostics; and genetics.

	Patient 1	Patient 2	Patient 3	Patient 4	Patient 5
**Age and Sex**	44 y/o, M	37 y/o, M	26 y/o, F	27 y/o, M	34 y/o, F
**Onset modality**	Subacute (3 weeks)	Subacute (5 weeks)	Acute (6–7 days)	Subacute (2 weeks)	Subacute (2 weeks)
**Onset symptoms**	Lower limb weakness; diffuse myalgia; drooping head	Exercise intolerance; diffused myalgia	Exercise intolerance; lower limb weakness	Exercise intolerance; lower limb weakness; fasting intolerance	Distal paresthesia followed by lower limb weakness
**Triggers**	Acute coronary syndrome	Not detected	Upper respiratory infection	Acute gastroenteritis	Acute gastroenteritis
**Onset CPK**	9 × n.v.	2.5 × n.v.	5 × n.v.	6 × n.v.	3.5 × n.v.
**CPK max value**	9 × n.v.	10 × n.v.	11 × n.v.	6 × n.v.	4 × n.v.
**Liver enzymes (AST, ALT)**	2 × n.v.	2 × n.v.	3 × n.v.	2.5 × n.v.	2 × n.v.
**LDH max value**	5 × n.v.	1.5 × n.v.	4 × v.n.	4.5 × n.v.	3.5 × n.v.
**Serum acylcarnitines**	Increase in C5 to C18 range	Increase in C5 to C18 range	Increase in C5 to C18 range	Increase in C5 to C18 range	Increase in C5 to C18 range
**Urinary organic acids**	Altered	Altered	Altered	Altered	Altered
**Electrophysiology (ENMG)**	Normal	Myopathic pattern with slight fibrillation activity	Myopathic pattern with fibrillation activity	Myopathic pattern with slight fibrillation activity	Normal EMG, sensory neuropathy
**Muscle MRI**	Bilateral marked and widespread edematous imbibition of several muscles (STIR+)	Edematous alterations, mainly posterior, in lower limbs (STIR+)	Not performed	Not performed	Not performed
** Genetic variants **	*ETFDH* gene homozygous variants c.1204A > G	*ETFDH* gene pathogenetic variant c.1852T > C	*ETFDH* gene compound heterozygosity c.358G > C/c.814G > A	*ETFDH* gene homozygous variants c.1286-15T > A	*ETFDH* gene pathogenetic variant c.406-2A > G
** Protein variant **	p.Thr402Ala	p.Ter618Gln	p.Asp120His/p.Gly272Arg	Intronic variant	Splicing variant

## Data Availability

The data is contained within the article.

## Abbreviations

The following abbreviations are used in this manuscript:

ACMG	American College of Medical Genomics
ALT	Alanine Transaminase
AST	Aspartate Transaminase
ATP	Adenosine Triphosphate
CNVs	Copy Number Variations
CoA	Coenzyme A
COMBI	Combined Stain COX-SDH
CoQ10	Coenzyme Q10
COX	Cytochrome C Oxidase Stain
CPK	Creatine Phosphokinase
CSF	Cerebrospinal Fluid
DBS	Dry Blood Spot
DNA	Deoxyribonucleic Acid
ETF	Electron Transfer Flavoprotein
ETFA	Electron Transfer Flavoprotein Subunit Alpha
ETFB	Electron Transfer Flavoprotein Subunit Beta
ETFDH	Electron Transfer Flavoprotein Dehydrogenase
ETF-QO	Electron Transfer Flavoprotein–Ubiquinone Oxidoreductase
FAO	Fatty Acid Oxidation
FEV1	Forced Expiratory Volume in 1 Second
FFAs	Free Fatty Acids
FO	Myophosphorylase Stain
FVC	Forced Vital Capacity
GAII	Glutaric Acidemia Type II
GBS	Guillain–Barré Syndrome
HE	Hematoxylin and Eosin Stain
IVIG	Intravenous Immunoglobulin
LDH	Lactate Dehydrogenase
MADD	Multiple Acyl-CoA Dehydrogenase Deficiency
MRI	Magnetic Resonance Imaging
MUPs	Motor Unit Potentials
n.v.	Normal Value
NADH	Nicotinamide Adenine Dinucleotide Stain
NGS	Next-Generation Sequencing
PAS	Periodic Acid–Schiff Stain
PCR	Polymerase Chain Reaction
QH2	Ubiquinol
RNA	Ribonucleic Acid
ROS	Reactive Oxygen Species
RT-PCR	Reverse Transcriptase Polymerase Chain Reaction
SDH	Succinate Dehydrogenase
STIR	Short Time Inversion Recovery
